# Stable Reference Genes for RT‐qPCR in House Dust Mite *Dermatophagoides farinae* (Acari: Pyroglyphidae) at Different Developmental Stages

**DOI:** 10.1002/ece3.74601

**Published:** 2026-10-29

**Authors:** Feng Jiang, Mingxia Ge, Zeyu Jin, Haoting Zhou, Mengmeng Zhang, Tong Guan, Haoyuan Hu, Jianhai Yin, Ying Liu

**Affiliations:** ^1^ School of Basic Medical Sciences Wannan Medical University Wuhu China; ^2^ National Institute of Parasitic Diseases, Chinese Center for Disease Control and Prevention; Chinese Center for Tropical Diseases Research; National Key Laboratory of Intelligent Tracking and Forecasting for Infectious Diseases; NHC Key Laboratory of Parasite and Vector Biology; WHO Collaborating Centre for Tropical Diseases; National Center for International Research on Tropical Diseases, Ministry of Science and Technology Shanghai China; ^3^ School of Ecology and Environment, Collaborative Innovation Center of Recovery and Reconstruction of Degraded Ecosystem in Wanjiang Basin Co‐Founded by Anhui Province and Ministry of Education Anhui Normal University Wuhu China; ^4^ School of Laboratory Medicine Wannan Medical University Wuhu China

**Keywords:** *Dermatophagoides farinae*, different developmental stages, expression stability, house dust mite, reference gene

## Abstract

To investigate the growth and development mechanisms in *Dermatophagoides farinae*, it is essential to understand its gene expression profiles across different life stages, which are typically assessed by transcriptional profiling based on RT‐qPCR. Suitable reference genes are vital for ensuring reliable and accurate results; however, none have been established for this species across different developmental stages. Here, we analyzed expression profiles of ten frequently used reference genes (*RPL18*, *RPS18*, *RPS*9, *EF1A*, *RPS28*, *Actin*, *V‐ATPaseB*, *GAPDH*, *RPL32*, and *α‐tubulin*) to determine their stability across different developmental stages of *D. farinae*. Four algorithms (GeNorm, NormFinder, BestKeeper, and ΔCt) were employed to examine gene stability, and their results were subsequently integrated by RefFinder to yield a comprehensive assessment. The results indicated that *RPL18* and *RPS28* were the two top‐ranked stable reference genes, whereas *α‐tubulin* and *RPL32* showed the lowest stability. To confirm the robustness of the chosen reference genes, we assessed cytochrome *P450 3A8* expression at developmental stages of *D. farinae* using three independent biological replicates and three technical replicates per stage. Normalization against *RPL18* and *RPS28*, individually or combined, produced expression patterns consistent with previously reported developmental trends of cytochrome *P450* genes in mites and insects, namely higher expression in larvae and protonymphs, lower expression in later stages, and minimal expression observed in eggs. In contrast, unstable reference genes yielded inconsistent results, thereby confirming our findings. In conclusion, this study identifies two highly stable reference genes that will provide a robust foundation for precise gene expression normalization and functional studies in *D. farinae*.

## Graphical abstract

First systematic evaluation of reference genes across different developmental stages of *Dermatophagoides farinae*. RPL18 and RPS28 are identified as the two stable reference genes. Cytochrome P450 3A8 gene validates that these stable reference genes provide robust and consistent expression patterns.

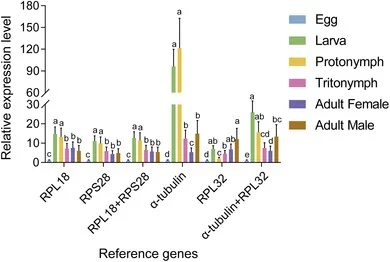

## 1 Introduction

*Dermatophagoides farinae* Hughes, 1961, house dust mite (HDM), which is found in domestic environments and stored products worldwide (Miller 2019; Vacante and Flechtmann 2024). Their allergens are one of the most common causes of allergic diseases, with more than 50% of inhalant allergy cases being associated with this type of mite (Sánchez‐Borges et al. 2017). Furthermore, HDMs are a significant pest of stored grains, capable of causing substantial economic losses (Savary et al. 2019). Nevertheless, its fundamental molecular biology remains poorly understood. A comprehensive understanding of these processes is essential for effective prevention and control, underscoring the urgency of functional investigations.

The life cycle of astigmatic mites generally consists of four stages: egg, larva, nymph, and adult (female and male) (Vacante and Flechtmann 2024). However, the nymphal stage varies among different taxa. Some groups exhibit three distinct nymphal instars, namely the protonymph, deutonymph (= hypopus), and tritonymph, such as certain taxa within Glycyphagidae (Corente and Knülle 2003), Histiostomatoidae (Klimov and Khaustov 2018), and Acaridae (Li et al. 2023). It should be noted that the hypopus stage is not an obligate phase in their development. It effectively withstands adverse environmental conditions (e.g., extreme temperatures, unfavorable humidity, and food scarcity), developing into a tritonymph once conditions become favorable. In contrast, the life cycle of *D. farinae* does not include a hypopus stage (Vacante and Flechtmann 2024).

Gene expression analysis is widely employed to investigate gene regulation and molecular mechanisms in various species. However, accuracy quantification of gene expression via RT‐qPCR hinges on the use of suitable reference genes to ensure proper normalization. Although several stably expressed reference genes are well‐documented in insects (Pan et al. 2015; Zhao et al. 2022) and other mites of the order Trombidiformes (Lee et al. 2022; Sun et al. 2010; Zhao et al. 2022), their expression stability has been shown to vary among species. For instance, *α‐tubulin* was identified as the most stable reference gene in *Tetrastichus hagenowii* (Dong et al. 2024), it proved to be the least stable in *Brevipalpus yothersi* (Rogerio et al. 2019). Similarly, *GAPDH* showed stable expression in *Rhipicephalus microplus* (Kim et al. 2023) but was unstable in *Varroa destructor* (Lee et al. 2022). Such inter‐species inconsistencies are largely driven by divergence in basal expression levels as well as stage‐ and tissue‐specific physiological and metabolic demands. Previous studies have identified *α‐tubulin* as the optimal reference gene for *D. farinae* under temperature stress conditions (Niu et al. 2019). Nevertheless, it has been well documented that the expression stability of reference genes can exhibit considerable variation across developmental stages and under diverse environmental stressors (Chang et al. 2017; Jiang et al. 2023; Wei et al. 2022).

In this study, the expression stability of ten candidate reference genes was evaluated across four developmental stages of *D. farinae* (egg, larva, nymph, and adult) using GeNorm, NormFinder, BestKeeper, and the ΔCt method, with a comprehensive ranking generated by RefFinder. The suitability of the selected genes was further validated by the cytochrome *P450 3A8* gene expression. This work aims to identify stable reference genes for the different developmental stages of *D. farinae*, to provide a technical foundation and reliable benchmarks for future functional studies, especially for the prevention and control of this type of mite based on RNA interference (RNAi) technology.

## 2 Materials and Methods

### 2.1 Mite Materials

*Dermatophagoides farinae*, originally sampled from floor dust collected at a retail store in Wuhu City, Anhui Province, China (31°27′ N, 118°35′ E) in June 2017, were maintained under long‐term laboratory rearing conditions. The mites were kept in a culture medium consisting of 70% bran, 9% wheat flour, 9% corn flour, 5% wheat germ powder, 5% yeast powder, and 2% sucrose in an artificial climate chamber at a temperature of 25°C ± 1°C and a relative humidity of 75% ± 5%, and in continuous darkness (0 h light:24 h dark). Individuals were selected based on morphological characteristics from four different developmental stages: egg, larva, nymphs (protonymph and tritonymph), and adults (female and male) (Figure 1). Eggs were obtained by congregating female adults for oviposition, and two‐day‐old eggs were subsequently collected. All samples were washed twice with PBS and subsequently dried using filter paper. The samples were flash‐frozen in liquid nitrogen and stored at −80°C for further use. For each biological replicate, approximately 2000 eggs, 2000 larvae, 900 protonymphs, 500 tritonymphs, 300 females, and 1000 males were pooled for total RNA extraction, respectively. These quantities were determined based on preliminary experiments to ensure sufficient RNA yield for subsequent qPCR analysis. Three biological replicates were performed for each developmental stage.

**FIGURE 1:**
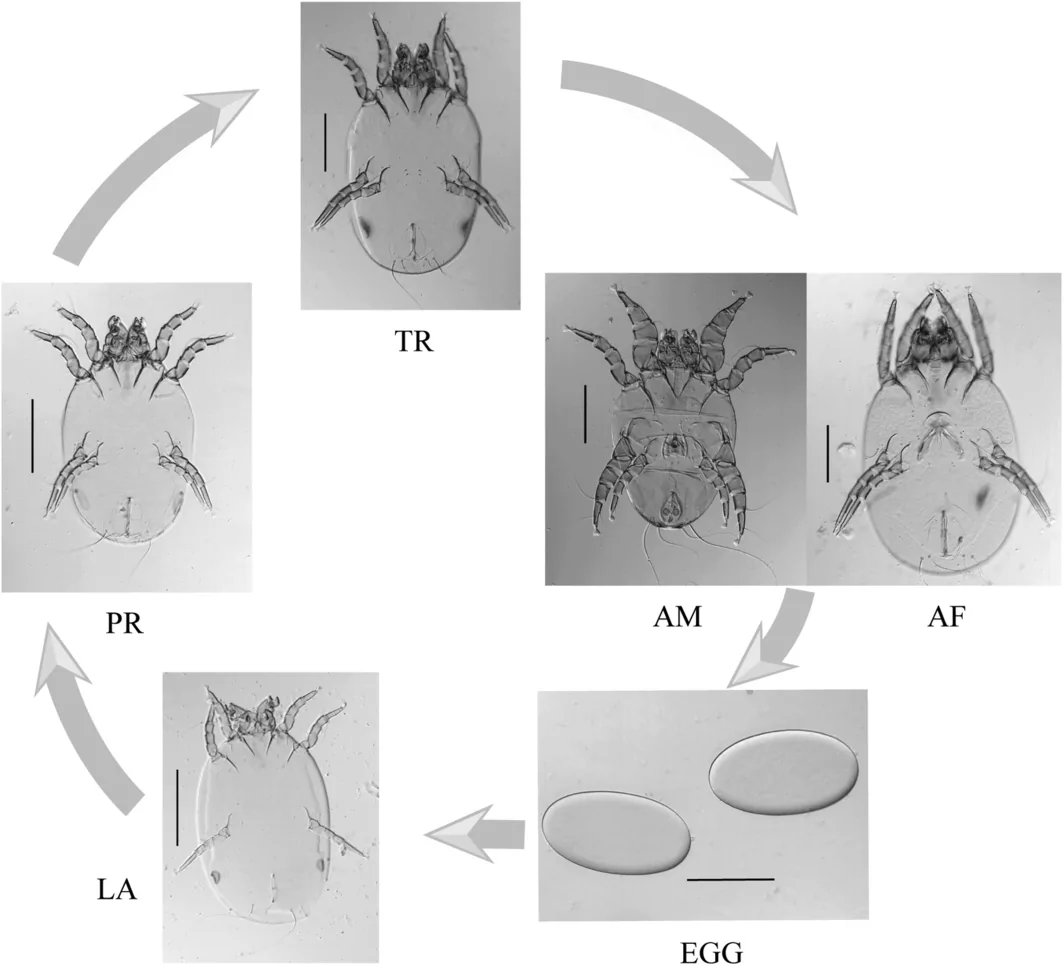
Morphological characteristics of *D. farinae* at different developmental stages. AF, Adult female; AM, Adult male; LA, Larva; PR, Protonymph; TR, Tritonymph. Scale bar = 100 μm. All photographs were taken by Feng Jiang.

### 2.2 Preparation of RNA and cDNA

The total RNA was extracted using the RNeasy Mini Kit (Qiagen, Germany). The concentration and purity of the extracted RNA were determined with a NanoDrop One spectrophotometer (Thermo Fisher Scientific, Waltham, MA, USA). RNA integrity was evaluated using a Bioanalyzer 2100 system (Agilent Technologies, CA, USA), and the degree of RNA degradation was assessed by 1% agarose gel electrophoresis. Then, 1 μg of total RNA was converted into cDNA through reverse transcription using the All‐in‐One First‐Strand Synthesis MasterMix (with dsDNase) (BestEnzymes Biotech Co. Ltd., China). The resulting cDNA was stored at −20°C for subsequent RT‐qPCR analysis.

### 2.3 Candidate Reference Genes Selection and Primer Design

Ten commonly used reference genes from insects and mites were selected as candidates for stability analysis (Table 1), namely 60S ribosomal protein L18‐like (*RPL18*) (Zhao et al. 2022), Actin (Rahman et al. 2021), V‐type proton ATPase subunit B (*V‐ATPaseB*) (Yang, Pan, Noland, et al. 2015), 40S ribosomal protein S9‐like (*RPS9*) (Rodrigues et al. 2014), elongation factor 1‐alpha (*EF1A*) (Niu et al. 2012), 40S ribosomal protein S18 (*RPS18*) (Zhou et al. 2025), glyceraldehyde‐3‐phosphate dehydrogenase 2‐like (*GAPDH*) (Bassan et al. 2017), 40S ribosomal protein S 28 (*RPS28*) (Yang et al. 2024), tubulin alpha chain (*α‐tubulin*) (Niu et al. 2019), and 39S ribosomal protein L32 (*RPL32*) (Zhang et al. 2015). The sequences of these homologous genes were obtained from the transcriptomic data of *D. farinae* (GCF_020809275.1, ASM2080927v1) available in the NCBI database. RT‐qPCR primers were designed and verified using the NCBI Primer‐BLAST tool, adhering to the following criteria: primer length of 18–25 bp, amplicon size ranging from 110 to 300 bp, Tm value of 60°C ± 3°C, a maximum ΔTm of 3°C between forward and reverse primers, and primer must span an exon‐exon junction (except for *EF1A*，*RPS18*，*RPS9*，and *RPL18*, which did not meet this criterion). The final primers were synthesized by General Biol Co. Ltd. (Chuzhou, China).

**TABLE 1:** Characteristics of primer and amplification for the 10 selected genes evaluated by RT‐qPCR in *D. farinae*.

Gene symbol	Gene name	GenBank accession number	Primer sequence (5′‐3′) forward/reverse	Exon boundary	Amplification efficiency (E%)	Correlation coefficient (*R* ^2^)	Product length (bp)	Melting temperature (TM) forward/reverse
*EF1A*	Elongation factor 1‐alpha	XM_047058349.1	AGAAGCTCAAGATATGGGCAA/GCAAAGCATGTTCACGGGTT	NO	105.12	0.9998	284	57.06/59.97
*RPS28*	40S ribosomal protein S 28	XM_047058558.1	ACTGTCAACTTGGATTACCCGA/CCACGGGCATACAATACAGA	Exon1: 2628759–2,629,153/Exon2: 2629216–2,629,394	99.51	0.9971	145	59.36/57.39
*RPS18*	40S ribosomal protein S18	XM_047058555.1	TTGCGTGAAGATTTGGAGCG/ACCACGTCGACCAGTTGTTT	NO	99.84	0.9947	111	59.48/60.11
*α‐tubulin*	Tubulin alpha chain	XM_047060774.1	AGGCTTCATTGCTGTCCCTT/CGCATTTTCGGTTACGGGTAT	Exon 1: 4323320–4,323,527/Exon 2: 4321641–4,323,203	100.26	0.9984	162	59.59/59.06
*Actin*	Actin	XM_047062445.1	CACGTCATCAGGGCGTCAT/GGGCTTCGGTCAAGAGAACA	Exon 1: 7435357–7,435,728/Exon 2: 7433806–7,435,284	99.94	0.997	213	60.15/59.97
*RPS9*	40S ribosomal protein S9‐like	XM_047056653.1	TCGTAAAGCTGCCCGTGAAT/ACGGATCAACACTCGTGCAT	NO	102.35	0.9995	235	60.04/60.04
*GAPDH*	Glyceraldehyde‐3‐phosphate dehydrogenase 2‐like	XM_047056629.1	GTGTACCTGTTCCGGACGTT/TCCGATGATACGACCTCTTCA	Exon 2: 299840–300,704/Exon 3: 299254–299,744	104.47	0.9979	157	59.97/58.07
*V‐ATPaseB*	V‐type proton ATPase subunit B	XM_047053991.1	ATGCGATGGCTGTTACTCGT/GGTGTACGTAAGATATCGCCTGT	Exon 1: 11570685–11,571,079/Exon 2: 11571140–11,572,537	94.81	0.9983	274	59.82/60.01
*RPL32*	39S ribosomal protein L32	XM_047053324.1	CTAAATCGTGCTCCACCAGC/GGCACACCCCATAATAATCCA	Exon 1: 1262194–1,262,355/Exon 2: 1,261,524–1,262,119	95.44	0.9975	119	58.99/57.76
*RPL18*	60S ribosomal protein L18‐like	XM_047057230.1	CGCTTCGACGATTGGTTCG/TTAGAATACGAGCACGGGCA	NO	97.21	0.9992	159	59.66/59.18

### 2.4 RT‐qPCR Analysis

All RT‐qPCR reactions were conducted on a LightCycler 96 System (Roche, Switzerland) using Taq SYBR Green qPCR Premix (Universal) (BestEnzymes Biotech Co. Ltd., China) following the manufacturer's recommended procedure. Each qPCR reaction (20 μL) was prepared using 0.2 μL of cDNA template, 10 μL of Taq SYBR Green qPCR Premix, 0.4 μL each primer (10 μM), and 9 μL of nuclease‐free water to reach the final volume. The thermal cycling conditions were as follows: an initial denaturation at 95°C for 30 s, after which 40 amplification cycles were performed, consisting of 10 s at 95°C and 30 s at 60°C. Melting curve analysis was carried out using the instrument's default program. For each of the six developmental time points (egg, larva, protonymph, tritonymph, female, and male) of *D. farinae*, all ten candidate reference genes were assessed using three independent biological replicates, each with three technical repeats, resulting in a total of 3 biological × 3 technical × 6 time points × 10 genes qPCR reactions. A serial dilution of cDNA (1×, 5×, 25×, 125×, 625×) from female samples was used to generate standard curves for calculating amplification efficiency and correlation coefficients, and determined using the formula %*E* = (10^(−1/slope)^–1) × 100% (Radonic et al. 2004).

### 2.5 Data Analysis

The Ct (Cq) values of the selected candidate reference genes were examined at different developmental stages. The stability and comprehensive ranking of candidate reference genes were evaluated using the RefFinder web‐based tool (http://blooge.cn/RefFinder/, accessed on 17 September 2025), which integrates four complementary algorithms: GeNorm, NormFinder, BestKeeper, and the ΔCt method. GeNorm evaluates gene expression stability (*M* value) by calculating the average pairwise variation of each candidate gene relative to all other candidates. Generally, a gene with an *M* value below 1.5 is considered to be stable. To determine the optimal number of reference genes required for accurate normalization, GeNorm calculates the pairwise variation (*V*_*n*/*n*+1_) between sequential normalization factors (NF_*n*_ and NF_*n*+1_). A cut‐off value of 0.15 was applied, indicating that the addition of the (*n* + 1)th gene does not significantly improve normalization stability when *V*_*n*/*n*+1_ falls below this threshold (Vandesompele et al. 2002). NormFinder assesses the stability of candidate genes by calculating both intra‐ and inter‐group variations for each gene, with lower (SV) indicating greater gene stability (Andersen et al. 2004). BestKeeper calculates the standard deviation (SD) and coefficient of variation (CV) across different samples; an SD < 1.0 indicates stable expression of the candidate reference gene (Pfaffl et al. 2004). The ΔCt method calculates the SD of ΔCt values for each gene across all samples, and the gene with the smallest SD is considered the most stable (Silver et al. 2006). The results from these four algorithms were integrated via RefFinder (Xie et al. 2023) to comprehensively rank the stability of candidate reference genes.

### 2.6 Suitability Validation

Cytochrome P450 monooxygenases play a critical role in xenobiotic metabolism and are expressed across the developmental stages in mites (Vlogiannitis et al. 2021; Yang, Pan, Liu, and Zhou 2015). To validate the reliability of the newly identified reference genes, we quantified the relative expression of cytochrome *P450 3A8* (GenBank: XM_047059725.2) in *D. farinae* using the 2^−ΔΔCt^ method (Livak and Schmittgen 2001). Expression levels were normalized using both stable and unstable reference genes, either individually or in combination. The counts of biological and technical replicates were consistent with those mentioned above. Gene expression data were first conducted to assess normality and variance homogeneity using the Shapiro–Wilk and Levene's tests in SPSS 27.0 (IBM Corp., USA). Normality and homogeneity of variance of the residuals were tested using Shapiro–Wilk and Levene's tests, respectively. Genes meeting both assumptions were analyzed by one‐way ANOVA with Tukey's HSD post hoc test, whereas those with significant variance heterogeneity were analyzed using Welch's ANOVA followed by Games–Howell post hoc test. All ANOVA and post hoc tests were performed on R platform (R version 4.3.1).

## 3 Results

### 3.1 Primer Specificity and PCR Amplification Efficiency

RNA extracted from 18 samples representing four developmental stages of *D. farinae* was used for qPCR experiments. The quality of all RNA samples was confirmed by spectrophotometric analysis (A260/A280 ratios between 1.9 and 2.1; A260/A230 ratios exceeding 2.0) and RIN values ranging from 6.2 to 8 (Table S1). All ten candidate genes displayed distinct single peaks (Figure S1), with amplification efficiencies ranging from 94.81% to 105.12% and correlation coefficients between 0.9947 and 0.9998 (Table 1).

### 3.2 Cq Values for Candidate Reference Genes

For RT‐qPCR amplification, 0.2 μL of cDNA was used per reaction. A lower Cq value indicates higher gene expression, whereas a higher Cq value corresponds to lower expression levels. Across all samples, the mean Cq values ranged from 18.29 ± 0.57 for *RPS18* to 23.42 ± 0.64 for *RPS28*. The coefficient of variation (CV) of gene expression can serve as a preliminary indicator of gene stability. Among the ten candidate reference genes evaluated, *RPL18* exhibited the smallest variation (2.7%), while *α‐tubulin* showed considerably higher variability (8.34%). These initial findings suggest that *RPS28*, *RPS18*, and *RPL18* are relatively stable and expressed at high levels (Figure 2, Table 2, Table S2).

**FIGURE 2:**
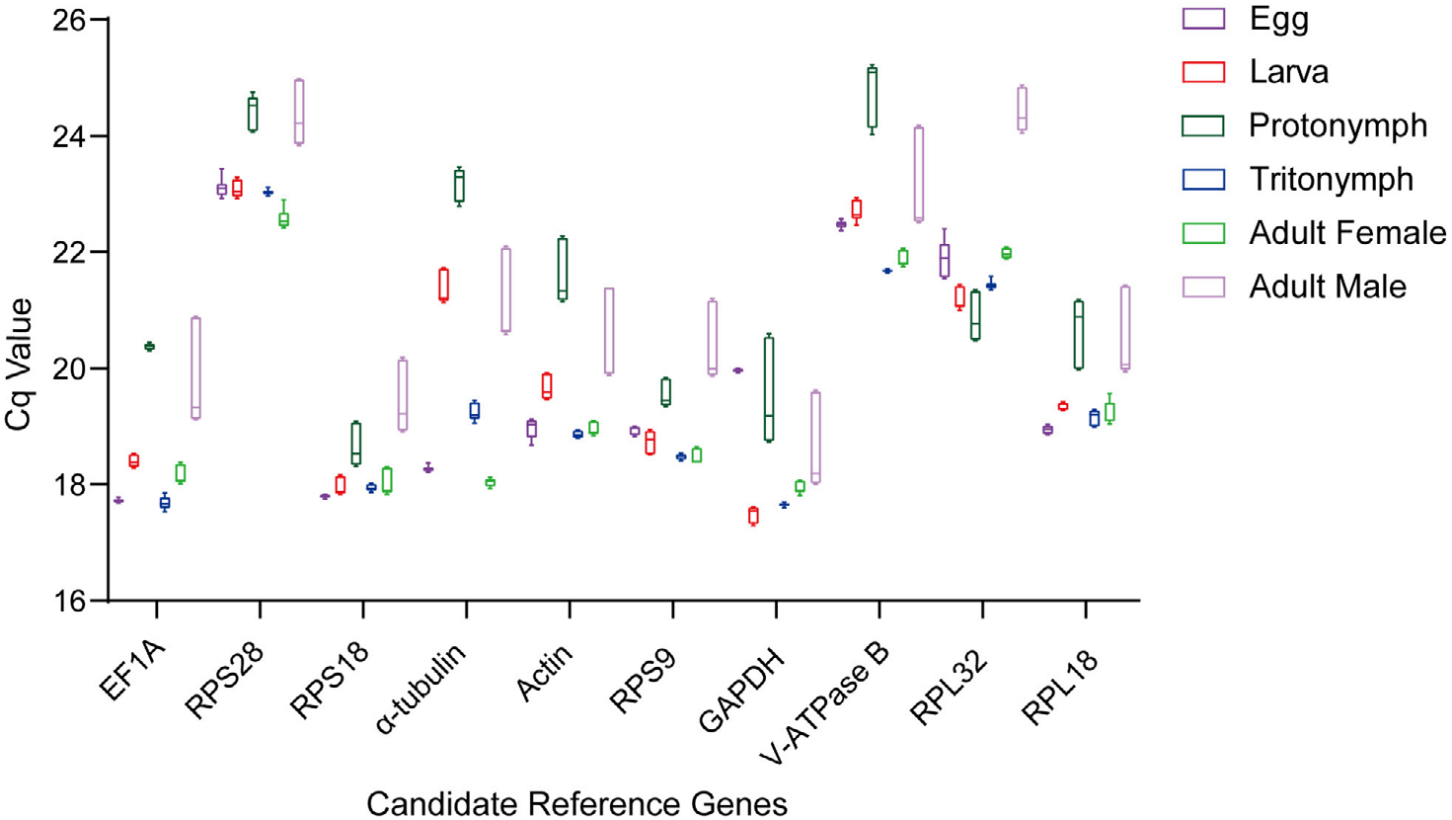
Box plots of Cq values for the ten candidate reference genes across different developmental stages of *D. farinae* (egg, larva, protonymph, tritonymph, adult female, and adult male). The whiskers denote the maximum and minimum values. The box encompasses the interquartile range (25% to 75%), with the central line indicating the median. No outliers were detected. Each box represents three biological replicates, each with three technical replicates.

**TABLE 2:** The stability profiles of the ten proposed reference genes in *D. farinae* were assessed using four distinct computational approaches.

Rank	GeNorm		Normfinder		BestKeeper			∆Ct	
	Gene	M	Gene	SV	Gene	SD	CV	Gene	SD
1	*RPL18*	0.311	*RPS28*	0.170	*RPS18*	0.49	2.70	*RPS28*	0.71
2	*RPS18*	0.311	*RPL18*	0.196	*RPS9*	0.57	3.00	*RPL18*	0.72
3	*RPS9*	0.359	*RPS9*	0.280	*RPL18*	0.60	3.06	*RPS9*	0.75
4	*RPS28*	0.374	*EF1A*	0.340	*RPS28*	0.64	2.72	*Actin*	0.77
5	*EF1A*	0.460	*Actin*	0.361	*V‐ATPaseB*	0.84	3.71	*EF1A*	0.77
6	*Actin*	0.496	*RPS18*	0.395	*RPL32*	0.86	3.93	*RPS18*	0.78
7	*V‐ATPaseB*	0.541	*V‐ATPaseB*	0.517	*Actin*	0.86	4.35	*V‐ATPaseB*	0.85
8	*GAPDH*	0.672	*GAPDH*	1.048	*GAPDH*	0.92	4.99	*GAPDH*	1.22
9	*RPL32*	0.806	*RPL32*	1.293	*EF1A*	0.93	4.96	*RPL32*	1.39
10	*α‐tubulin*	0.946	*α‐tubulin*	1.431	*α‐tubulin*	1.68	8.34	*α‐tubulin*	1.51

### 3.3 Evaluation of the Stability

The consistency of the potential reference genes was assessed through four different approaches: GeNorm, NormFinder, BestKeeper, and the ∆Ct method. Based on the results from GeNorm and BestKeeper, the top three genes with the highest expression stability were identified as *RPL18*, *RPS18*, and *RPS9*. In contrast, NormFinder and the ∆Ct method revealed *RPS28*, *RPL18*, and *RPS9* as the top three most stable genes. The *α‐tubulin* consistently exhibited the lowest stability across all four methods (Table 2). In the GeNorm analysis, the calculated average stability (*M*) values for all ten candidate genes were below the threshold of 1.5 (except *α‐tubulin*) (Figure 3). In the BestKeeper analysis, the standard deviation (SD) values of nine candidate reference genes were below 1.0, with the exception of *α‐tubulin* (Table 2). According to the overall ranking generated by RefFinder, the genes were ordered from highest to lowest stability as follows: *RPL18* > *RPS28* > *RPS18* > *RPS9* > *Actin* > *EF1A* > *V‐ATPaseB > GAPDH* > *RPL32* > *α‐tubulin* (Table 3).

**FIGURE 3:**
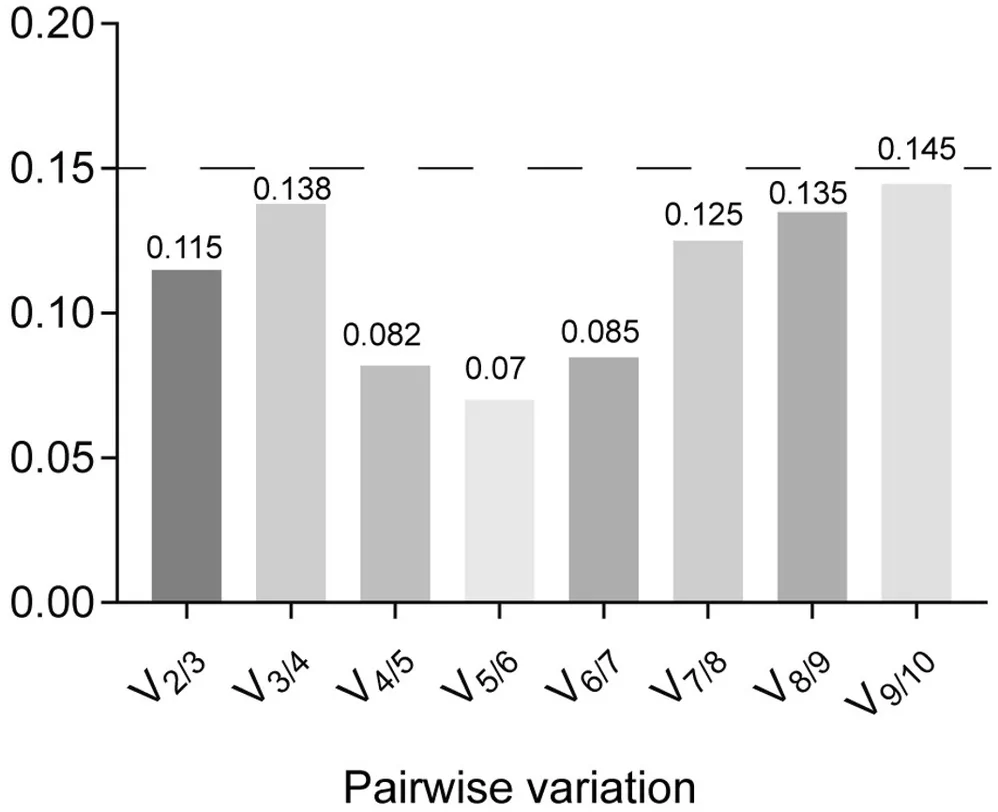
Pairwise variation (*V*) was employed to identify the most suitable number of reference genes for different developmental stages of *D. farinae*, applying a cut‐off value of 0.15.

**TABLE 3:** Assessment and ranking of ten reference gene candidates using RefFinder.

Rank	Candidate genes	Ranking value^a^
1	*RPL18*	1.86
2	*RPS28*	2.00
3	*RPS18*	2.45
4	*RPS9*	2.71
5	*Actin*	5.18
6	*EF1A*	5.48
7	*V‐ATPaseB*	6.44
8	*GAPDH*	8.00
9	*RPL32*	8.45
10	*α‐tubulin*	10.00

^a^ The ranking value is the geometric mean of the ranks obtained from the four algorithms (geNorm, NormFinder, BestKeeper, and ΔCt method); lower values indicate higher stability.

### 3.4 Identification of the Optimal Count of Reference Genes

GeNorm analysis was performed to assess the pairwise variation (*V*) values of the ten candidate genes at four developmental stages of *D. farinae*. In this study, the *V*_2/3_ value (0.115) was less than the threshold of 0.15, and this criterion was consistently maintained for all subsequent pairwise comparisons (Figure 3). Therefore, to ensure accurate normalization of target genes when analyzing gene expression across the different developmental stages, two reference genes (*RPL18* and *RPS28*) were determined to be optimal for *D. farinae* based on the overall ranking generated by RefFinder (Table 3).

### 3.5 Gene Verification

To assess the stability of the selected reference genes, the expression of cytochrome *P450 3A8* gene was analyzed under different developmental stages of *D. farinae*. When the two most stable reference genes were used individually or in combination (*RPL18*, *RPS28*, or *RPL18* + *RPS28*), the normalized expression levels of the target gene demonstrated a remarkable degree of consistency (Figure 4). Under these normalization strategies, the cytochrome *P450 3A8* expression was relatively higher in larva and protonymph, with no statistically significant difference between these two stages, whereas expression levels were lower in tritonymph and adults (female and male), and lowest in eggs. Relative to eggs, expression levels increased by 10.9‐ to 14.6‐fold in larvae, 9.7‐ to 11.9‐fold in protonymphs, 1.7‐ to 6.9‐fold in tritonymphs, and 4.3‐ to 7.6‐fold in adults. Significant differences were observed between larva/protonymph and tritonymph/adult stages, with fold differences ranging from 2.4‐ to 2.5‐fold, as well as between egg and all other stages (Figure 4, Tables S3, S4). The correlation coefficients among the three normalization methods ranged from 0.9709 to 0.9935, and the coefficients of variation (CV) across developmental stages remained relatively stable, ranging from 8.35% to 22.55% (Tables S5, S6).

**FIGURE 4:**
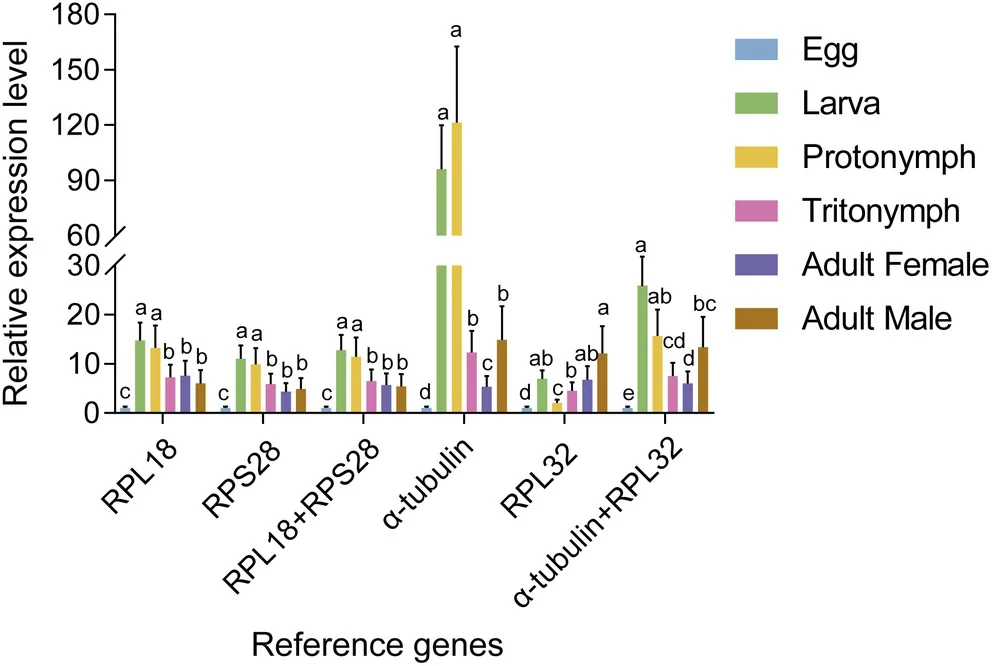
Assessment of the cytochrome *P450 3A8* gene expression across different developmental stages of *D. farinae* normalized with the top‐ranked (*RPL18*, *RPS28*, and *RPL18* + *RPS28*) and the least stable (*α‐tubulin*, *RPL32*, and *α‐tubulin* + *RPL32*) reference genes. The egg stage was used as the calibrator for relative quantification (set to 1). Statistically significant differences among stages are indicated by different lowercase letters.

In contrast, when the two least stable reference genes (*α‐tubulin*, *RPL32*, or *α‐tubulin* + *RPL32*) were used for normalization, cytochrome *P450 3A8* expression exhibited highly unstable patterns (Figure 4). When normalized to *α‐tubulin*, expression was highest in protonymphs and larvae, followed by adult males and tritonymphs, whereas adult females and eggs showed significantly lower expression. In contrast, *RPL32*‐based normalization yielded relatively high expression in adult males, larvae, and adult females, with lower levels in tritonymphs, protonymphs, and eggs. Combined normalization with *α‐tubulin* and *RPL32* produced yet another pattern, although expression remained relatively high in larvae and protonymphs. These inconsistent patterns highlight the substantial influence of reference gene selection on the interpretation of cytochrome *P450 3A8* expression across developmental stages (Figure 4, Tables S3, S4). The correlation coefficients among the three unstable normalization methods were low, ranging from −0.5346 to 0.7487, and the CV values across developmental stages were highly variable, ranging from 8.42% to 115.03% (Tables S5, S6).

## 4 Discussion

Elucidating the growth and development mechanisms of mites, and thereby identifying key genes or physiological processes involved in their development, can enable the establishment of efficient and precise control strategies. To achieve accurate gene expression profiling across developmental stages, reliable normalization targets are indispensable. Prior to this study, reference gene validation in HDMs was restricted to temperature stress conditions (Niu et al. 2019), with no validated targets available across different developmental stages. Therefore, we performed a systematic assessment of ten candidate reference genes across different developmental stages of *D. farinae* to establish a validated normalization baseline for future functional and developmental studies.

The reliability of RT‐qPCR results is contingent upon several critical factors, including RNA quantity, primer amplification efficiency, and the reference genes (Shi, Yang, et al. 2016). Accurate RT‐qPCR analysis is contingent upon both RNA integrity and accurately determined primer efficiency (Bustin et al. 2009). In this study, all RNA samples exhibited OD260/280 ratios between 1.9 and 2.1, RIN values ranging from 6.2 to 8, and the amplification efficiencies ranged from 94.81% to 105.12% (all *R* ^2^ > 0.9947). Consequently, the quality of both the RNA and primers used in this investigation was deemed adequate for subsequent experimental procedures.

Across various developmental stages of *D. farinae*, *RPL18* and *RPS28* emerged as the most suitable reference genes among ten candidates, whereas *α‐tubulin* and *RPL32* proved to be the least appropriate. *RPL18*, a key component of the large ribosomal subunit, is involved in nearly all protein biosynthesis processes (Leidig et al. 2014). It has been established as a stable reference gene in the developmental stages of *Bradysia odoriphaga* (Shi, Yang, et al. 2016) and *Cnaphalocrocis medinalis* (Zhao et al. 2022), the tissues of *Cimex lectularius* (Mamidala et al. 2011) and *Plutella xylostella* (You et al. 2018), and castes of *Solenopsis invicta* (Cheng et al. 2013). Similarly, *RPS28* has been previously established as a reliable reference gene under differential developmental conditions (Jia et al. 2019; Reddy et al. 2023). *α‐tubulin*, a major cytoskeletal component, was identified as the least stable reference gene across four computational methods (GeNorm, NormFinder, BestKeeper, and ∆Ct) and in the final RefFinder ranking in our study. This finding aligns with a report in *Schistosoma mansoni* , where *α‐tubulin* was also the least stable gene (Silveira et al. 2021). In contrast, it was proved to be the most suitable reference gene across distinct developmental stages in *Bactrocera tau* (Zhai et al. 2025) and *Drosophila suzukii* (Zhai et al. 2014), and under various temperature stress conditions in *D. farinae* (Niu et al. 2019). The divergent stability of α‐tubulin in *D. farinae* under temperature stress versus across developmental stages likely stems from distinct biological mechanisms. As an essential component of the cytoskeleton, *α‐tubulin* expression is tightly coupled with cellular proliferation, tissue differentiation, and biological remodeling. During the development of *D. farina* from egg to adult, drastic physiological and morphological transitions require rapid cytoskeletal reorganization, resulting in considerable fluctuations in *α‐tubulin* transcript levels. In contrast, under short‐term temperature stress, metabolic adjustments or heat‐shock responses may prevail without triggering the extensive structural remodeling or developmental cell division associated with life‐cycle transitions, thus allowing *α‐tubulin* expression to remain relatively stable. These findings demonstrate the species‐ and condition‐specific nature of reference genes, thereby supporting the view that extrapolating their reliability across species is inherently risky. Consequently, it is essential for researchers to perform appropriate validation before employing reference genes in their studies.

The cytochrome P450 enzymes constitute one of the most critical metabolic detoxification systems in insects and are also involved in regulating a variety of endogenous physiological processes (Feyereisen 1999; Lu et al. 2021). Expression analysis across different developmental stages of mites (Ding et al. 2013; Shi, Zhang, et al. 2016) and insects (Guo et al. 2016; Huang et al. 2012; Wang et al. 2015) revealed the highest expression level in larvae, followed by nymphs and adults, whilst the lowest expression was observed in eggs. In our study, the two most stable reference genes (*RPL18* and *RPS28*), whether used individually or in combination, yielded consistent results, with expression trends aligning with those observed for homologous genes in other mite or insect species. In contrast, when two unsuitable genes (*α‐tubulin* and *RPL32*) were employed for normalization, the results showed inconsistent trends and deviated from the expected pattern. Therefore, *RPL18* and *RPS28* are proposed as reliable reference genes for normalizing gene expression in the various developmental stages of *D. farinae*.

Although this work offers the first experimentally confirmed markers suitable for normalizing transcriptional profiling across distinct developmental stages of *D. farinae*, some limitations remain. The ten candidate genes, chosen for their common application in insects and other mites, are practical yet may not encompass all potential stable genes. Future transcriptome‐wide screening may identify novel and potentially more stable candidates. Furthermore, as the present study focused exclusively on developmental stages, the reliability of *RPL18* and *RPS28* under other physiological or environmental conditions, such as fluctuations in temperature and humidity, or changes in food sources, requires further investigation. Additionally, the *D. farinae* used in this study was collected in 2017 and maintained under long‐term laboratory culture for numerous generations; laboratory adaptation may alter its baseline gene expression and stability profiles relative to natural wild populations. Future evaluations of freshly sampled wild populations will help confirm the broad generalizability of these target reference genes. In addition, no‐template controls and no‐reverse transcription controls were not included in the qPCR assays, which represents a limitation relative to full MIQE compliance (Bustin et al. 2009). Furthermore, the number of pooled individuals varied considerably across developmental stages, ranging from 300 for adult female to 2000 for egg. Although three biological replicates were performed for each stage, this disparity in pool size may limit the biological representativeness for smaller‐bodied or rarer stages, where inter‐individual variation may be less adequately captured. Nevertheless, the high consistency among biological replicates supports the reliability of our findings. Finally, as the investigation was confined to a single species, the findings may not be representative of all mites within the diverse cohort Astigmatina. Future studies will include representative species from various families and genera to identify stable reference genes applicable at the superfamily or family level within Astigmatina.

In conclusion, this study investigated the stability of ten reference genes across four developmental stages of *D. farinae*. Under the experimental conditions of this work, our findings demonstrate that *RPL18* and *RPS28* are suitable for normalizing gene expression, whether employed individually or in combination. This study provides a valuable resource and specific option for future gene expression analysis, contributing to a more accurate understanding of the molecular mechanisms underlying the development of *D. farinae*.

## Author Contributions

**Feng Jiang:** conceptualization (equal), data curation (lead), formal analysis (lead), investigation (equal), methodology (equal), resources (equal), validation (lead), visualization (equal), writing – original draft (lead), writing – review and editing (equal). **Mingxia Ge:** data curation (equal), formal analysis (equal), methodology (lead), validation (equal), visualization (lead). **Zeyu Jin:** investigation (lead), methodology (equal). **Haoting Zhou:** investigation (equal), methodology (equal). **Mengmeng Zhang:** investigation (equal), methodology (equal). **Tong Guan:** investigation (equal), methodology (equal). **Haoyuan Hu:** conceptualization (equal), methodology (equal). **Jianhai Yin:** conceptualization (lead), formal analysis (equal), funding acquisition (equal), project administration (lead), supervision (equal). **Ying Liu:** conceptualization (equal), formal analysis (equal), funding acquisition (equal), resources (lead), supervision (lead), writing – review and editing (lead).

## Funding

This work was supported by the NHC Key Laboratory of Parasite and Vector Biology (National Institute of Parasitic Diseases, Chinese Center for Diseases Control and Prevention), NHCKFKT2024‐9; The Doctoral Research Start‐up Fund of Wannan Medical College, WYRCQD2023011, WYRCQD2023019.

## Conflicts of Interest

The authors declare no conflicts of interest.

## Supporting information


**Table S1:** The RNA profiles extracted from four different developmental stages of *D. farinae*.
**Table S5:** Correlation coefficients for cytochrome *P450 3A8* gene expression normalized by stable and unstable reference gene combinations at different developmental stage of *D. farinae* (egg stage excluded).
**Table S6:** Coefficients of variation (CV, 100%) of cytochrome *P450 3A8* gene expression normalized by stable and unstable reference gene combinations at different developmental stage of *D. farina* (egg stage excluded).
**Figure S1:** Melting peaks of the candidate reference genes in different developmental stages of *D. farinae*.

**Table S2:** Cq values of 10 candidate reference genes in *D. farinae* samples.

**Table S3:** Results of Levene's test for homogeneity of variance in candidate reference genes.

**Table S4:** ANOVA and post‐hoc comparisons of candidate reference gene expression at different developmental stages.

## Data Availability

The data that supports the findings of this study are available in the Supporting Information of this article.
