# Supplementary material for: Stable Reference Genes for RT‐qPCR in House Dust Mite Dermatophagoides farinae (Acari: Pyroglyphidae) at Different Developmental Stages
Source: Ecol Evol. 2026 Oct 29;16(11):e74601. doi: 10.1002/ece3.74601 (PMC13717486; doi:10.1002/ece3.74601)
Supplement: Supplementary file 1 [file ECE3-16-e74601-s002.docx]

**TABLE S1** The RNA profiles extracted from four different developmental stages of *D. farinae*

| Samples | Concentration (ng/µL) | A260/A280 | A260/A230 | RIN |
| --- | --- | --- | --- | --- |
| Egg 1 | 216.9 | 2.10 | 2.36 | 6.7 |
| Egg 2 | 150.3 | 2.04 | 2.23 | 7.4 |
| Egg 3 | 182.1 | 2.05 | 2.35 | 7.2 |
| Larva 1 | 92.1 | 2.03 | 2.19 | 7.9 |
| Larva 2 | 137.7 | 2.08 | 2.19 | 6.5 |
| Larva 3 | 185.1 | 1.92 | 2.05 | 8 |
| Protonymph 1 | 114.8 | 2.06 | 2.08 | 6.9 |
| Protonymph 2 | 170.3 | 2.05 | 2.27 | 7.4 |
| Protonymph 3 | 134.8 | 2.08 | 2.18 | 6.7 |
| Tritonymph 1 | 131.0 | 2.03 | 2.36 | 7.6 |
| Tritonymph 2 | 166.5 | 2.04 | 2.40 | 6.9 |
| Tritonymph 3 | 169.2 | 2.09 | 2.00 | 7.1 |
| Adult Female 1 | 240.1 | 2.06 | 2.50 | 6.5 |
| Adult Female 2 | 534.5 | 2.04 | 2.36 | 7.8 |
| Adult Female 3 | 209.6 | 2.05 | 2.34 | 7.3 |
| Adult Male 1 | 94.6 | 2.09 | 2.19 | 6.2 |
| Adult Male 2 | 109.6 | 2.05 | 2.12 | 6.9 |
| Adult Male 3 | 104.0 | 2.06 | 2.08 | 6.4 |

**TABLE S5** Correlation coefficients for cytochrome *P450 3A8* gene expression normalized by stable and unstable reference gene combinations at different developmental stage of *D. farinae* (egg stage excluded)

|  | *RPL18* | *RPS28* | *RPL18*+*RPS28* | *α-tubulin* | *RPL32* | *α-tubulin*+  *RPL32* |
| --- | --- | --- | --- | --- | --- | --- |
| *RPL18* | 1 |  |  |  |  |  |
| *RPS28* | 0.9709 | 1 |  |  |  |  |
| *RPL18*+*RPS28* | 0.9919 | 0.9935 | 1 |  |  |  |
| *α-tubulin* | 0.9363 | 0.9428 | 0.9469 | 1 |  |  |
| *RPL32* | -0.5108 | -0.4681 | -0.4917 | -0.5346 | 1 |  |
| *α-tubulin*+  *RPL32* | 0.8110 | 0.8607 | 0.8434 | 0.7487 | 0.0449 | 1 |

**TABLE S6** Coefficients of variation (CV, 100%) of cytochrome *P450 3A8* gene expression normalized by stable and unstable reference gene combinations at different developmental stage of *D. farina* (egg stage excluded)

|  | LA | PR | TM | AF | AM |
| --- | --- | --- | --- | --- | --- |
| *RPL18, RPS28, RPL18* + *RPS28* | 11.85 | 12.17 | 8.29 | 22.55 | 8.35 |
| *α-tubulin, RPL32, α-tubulin* + *RPL32* | 89.19 | 115.03 | 39.07 | 9.45 | 8.42 |

**FIGURE S1** Melting peaks of the candidate reference genes in different developmental stages of *D. farinae*
